# The relationship between coffee intake, obstructive sleep apnea risk, and type 2 diabetes glycemic control, in Tabuk City, The Kingdom of Saudi Arabia: a case–control study

**DOI:** 10.1186/s13104-019-4838-3

**Published:** 2019-12-09

**Authors:** Mohammed Adam Ahmed Elnour, Abdulmoneim Ahmed Saleh, Mowffaq Mohammed Kalantan, Hyder Osman Mirghani

**Affiliations:** 1 Department of Internal Medicine, Faculty of Medicine, University of Tabuk, PO Box 3378, Tabuk, 51941 Saudi Arabia; 2 Department of Family and Community Medicine, Faculty of Medicine, University of Tabuk, Tabuk, Saudi Arabia

**Keywords:** Coffee intake, Obstructive sleep apnea risk, Daytime sleepiness, Saudi Arabia

## Abstract

**Objectives:**

The study aimed to assess the relationship between coffee intake, obstructive sleep apnea risk (OSA), and glycemic control among patients with diabetes mellitus.

**Results:**

There were 110 patients with diabetes and 96 healthy control subjects (matched for age and sex) attending a diabetes center زinTabuk, Saudi Arabia during the period from June 2018–October 2019. Stop-Bang questionnaire was used to assess OSA risk, and Epworth Sleepiness Scale to investigate daytime sleepiness. OSA risk and daytime sleepiness were higher among patients with diabetes compared to controls (4.34 ± 1.61 vs. 2.86 ± 1.24, and 8.31 ± 4.40 vs. 6.39 ± 3.70 respectively, P < 0.5), while coffee consumption was not (4.64 ± 3.95 vs. 3.45 ± 3.06, P > 0.05). Women with diabetes were younger with short duration since the diagnosis of diabetes and consumed less coffee compared to men, P < 0.5. A negative correlation was found between coffee consumption and the duration of diabetes, while no correlation was found between coffee intake, the glycated hemoglobin, OSA risk, sex, and daytime sleepiness. Daytime sleepiness and OSA risk were commoners among patients with diabetes, they were not correlated with coffee consumption which was negatively correlated with the duration since diabetes diagnosis. Further larger multi-center studies investigating coffee intake among patients newly diagnosed with diabetes are recommended.

## Introduction

Diabetes mellitus is highly prevalent worldwide. Currently, 350 million are estimated to suffer from the disease and 8 million people had prediabetes [1–4]. The Kingdom of Saudi Arabia is among the countries with the highest prevalence (21.1%) [5, 6].

Obstructive sleep apnea (OSA) andHabitual snoring are highly prevalent in particular among patients with diabetes mellitus and glucose intolerance [7, 8]. In addition to the worsening of diabetes control, obstructive sleep apnea leads to impaired memory, cardiovascular dysfunction, and excessive daytime sleepiness. In spite of being the most used psychoactive drug affecting both sleep–wake cycles, caffeine effects on obstructive sleep apnea are largely unknown [9]. The link between coffee consumption to mitigate daytime sleepiness or as a habit is unclear.

Previous literature observed the protective effects of coffee intake on diabetes risk. [10, 11]. While others concluded mixed results [12], a prospective study assessed coffee intake to meal concluded the inverse relationship between coffee intake at lunch and diabetes risk [13]. Regarding coffee intake and obstructive sleep apnea, the available data showed conflicting findings: A community-based study conducted in the United States of America concluded that Caffeinated soda, but not tea or coffee intake was independently associated with sleep-disordered breathing severity [14]. Pinheiro et al. [9] conducted a 20-year retrospective study and showed that there is no association between coffee intake and the development of obstructive sleep apnea, but may be correlated with the associated sleepiness and severity of the syndrome.

A review of epidemiological studies and clinical trials reported contradicting results [15]. Diabetes mellitus and obstructive sleep apnea are highly prevalent worldwide and when present together they exacerbate each other deleterious consequences. The earlier detection and treatment of OSA among patients with type 2 diabetes could substantially improve diabetes control and cost. Furthermore, both diseases are largely preventable through lifestyle management. There is a misconception regarding coffee intake even among treating physicians especially in patients with diabetes, hypertension, and cardiovascular disease.

Coffee is the readily available and cheap product if proved to be effective could be of great benefit in terms of cost and improvement in the quality of life among patients with type 2 diabetes and obstructive sleep apnea. We aimed to assess the relationship between coffee consumption, obstructive sleep apnea risk, and glycemic control among patients with type 2 diabetes in Tabuk City, Saudi Arabia.

## Main text

### Subjects and methods

#### Research design

A case–control study was conducted at the Diabetes Center in Tabuk King Fahad Specialist Hospital during the period from June 2018 to October 2019. One hundred and ten participants with type 2 diabetes and 96 healthy controls (enrolled from the co-patients to minimize socioeconomic differences) were included in the study. Patients on sleep medications, comorbidities including acromegaly, hypothyroidism, hormone replacement therapy, postoperative cardiac patients and those on steroids, alcoholic, and liver disease, rheumatic and psychological disorders were not included.

#### Study population and sampling

Subjects with the diagnosis of type 2 diabetes at the diabetes center in King Fahad Specialist Hospital were approached. The diabetes center is the only center in Tabuk and serves patients referred from the primary care centers and other general Hospitals in Tabuk. The sample size was calculated using the formula: n = Z^2^ P Q/d^2^ where Z = 95% confidence (1.96), P = prevalence of diabetes mellitus in KSA (3), and Q = 100. The patients were interviewed by the investigators (face to face) using a structured questionnaire that inquires about demographic data, coffee consumption frequency and amount (cups/day), and physical exercise. The Arabic versions of the STOP-BANG and Epworth Sleepiness Scales were used to assess the risk of obstructive sleep apnea and daytime sleepiness respectively. The (ESS), a self- reported scale for testing daytime sleepiness has been previously validated for use in Arab countries [16, 17]. The questionnaire inquire about if dose or feel asleep in eight different situations: while watching TV, sitting and reading, sitting inactive in public places, as a passenger in a car for 1 h without a break, lying down to rest in the afternoon when circumstances permit, sitting and talking to another person, sitting quietly after a lunch without ethanol consumption, and in a car, while stopped for a few minutes in traffic. The total score is 24, each question has 4 choices scoring from 0 to 3 with 0 = no and 3 = severe tendency to dose. A score of ≥ 10 out of 24 is considered as daytime sleepiness. The STOP-BANG questionnaire is concise, easy to use, and accurate tool for assessing obstructive sleep apnea. The questionnaire has been previously validated as an excellent screening tool for obstructive sleep apnea in sleep clinics for different populations [18]. The scale consists of eight yes/no items (dichotomous) related to the risk of obstructive sleep apnea, namely: snoring, tiredness, observed apnea, high blood pressure, body mass index, age, neck circumference, and gender [19]. The total score ranges from zero to eight. With a sensitivity of 93% and 100% to detect moderate to severe OSA (apnea–hypopnea index [AHI] > 15) and severe OSA (AHI > 30) respectively at score of ≥ 3 [17, 18]. The Arabic version of the questionnaire showed high validity and reliability in the Arab World [20]. Neck circumference (NC) is abnormal if > 37 cm and 34 cm in men and women respectively [21, 22].

Weight, height, and Body Mass Index (BMI) were measured using the formula: Weight in Kg/height in (meters). The most recentHbA1c was collected from the patient’s records. All participants were invited to sign a written informed consent, and ethical approval was obtained from the ethical committee of the University of Tabuk (Ref. UT-69-18-2018, date 25/10/2018.

Data were exported to the Statistical Package for Social Sciences (IBM, version 20, New York) for the analysis. Descriptive and summary statistics were carried out to describe study participants according to different characteristics and proportions were computed to find out the relationship between coffee intake, obstructive sleep apnea, and type 2 glycemic control. A P-value of < 0.05 was considered significant.

### Results

#### Obstructive sleep apnea risk, daytime sleepiness, body mass index, and adherence to regular exercise among the study group

Out of 110 patients with diabetes mellitus and ninety-six control subjects matched for age, sex, and level of exercise, patients with diabetes had a higher body mass index compared to their counterparts, a high statistical difference was found between patients with diabetes and control subjects regarding OSA risk (88.5% vs. 56.5%, P = 0.000, no statistical difference was evident regarding the daytime sleepiness, P = 0.109) Table 1.

**Table 1:** General characters of the study group

Character	DM	Control	P-value	95% CI
Age (mean ± SD	51.18 ± 10.79	49.32 ± 5.51	0.272	
Sex			0.252	0.283–1.39
Males	50 (47.2%)	54 (62.7%)		
Females	56 (52.8%)	38 (37.3)		
Level of exercise	34 (31.5%)	30 (32.6%)	0.649	
Obesity	52 (50%)	22 (23.9%)	0.008	
OSA risk (STOP-BANG)	92 (88.5%)	52 (56.5%)	< 0.001	2.10–16.54
Daytime sleepiness	38 (36.5%	20 (21.7%)	0.109	0.19–1.18

Independent samples t-test and Chi square test

#### Epworth sleepiness score, STOP-BANG score, and coffee intake among the study group

Table 2 showed a comparison between patients with diabetes and controls in which: OSA risk, and daytime sleepiness were higher among patients with diabetes compared to controls (4.34 ± 1.61 vs. 2.86 ± 1.24, and 8.31 ± 4.40 vs. 6.39 ± 3.70 respectively, P = 0.21, and 0.000 respectively), while coffee (Arabic coffee without sugar) consumption was not (4.64 ± 3.95 vs. 3.45 ± 3.06, P ≥ 0.100).

**Table 2:** A comparison between patients with diabetes and control subjects regarding Epworth sleepiness score, Stop-Bang score, and coffee intake

Character (mean ± SD)	Diabetes	Control	P-value
Daytime sleepiness	8.31 ± 4.40	6.39 ± 3.70	0.021
STOP-BANG score	4.34 ± 1.61	2.86 ± 1.24	< 0.001
Coffee intake(cups/day)	4.64 ± 3.95	3.45 ± 3.06	0.100

Independent samples t-test

#### The correlation of coffee intake with different characters among patients with diabetes

In the present survey, interestingly coffee intake was found to be negatively correlated with diabetes duration (Wald = 3.85, and P = 0.50), no significant statistical correlations were reported regarding glycemic control (Wald = 0.002, P = 0.962), and obstructive sleep apnea risk (Wald  = 0.077, P = 0 .781) Table 3. Illustrated the correlation of coffee consumption and different parameters among patients with diabetes.

**Table 3:** The relationship of coffee intake to Epworth sleepiness score, Stop-Bang score, sex, HbA1c, and the duration since diabetes diagnosis

Character	B	SE.	Wald	df	Sig.	Exp. (B)	95% CI for Exp.
Daytime sleepiness	− 0.114	0.716	0.025	1	0.874	0.893	0.220–3.630
STOP-BANG score	− 0.298	1.071	0.077	1	0.781	0.743	0.091–6.061
Sex	0.019	0.759	0.001	1	0.980	1.020	0.231–4.509
HbA1c	0.009	0.198	0.002	1	0.962	1.009	0.684–1.489
Diabetes duration	−0.092	0.047	3.856	1	0.050	0.912	0.832–1.000
Constant	0.938	2.192	0.183	1	0.669	2.555	

Logistic regression analysis

### Discussion

In the present study, obstructive sleep apnea risk among patients with diabetes was 88.5% and was significantly higher than control subjects, the present data are similar to a study conducted in Taif Saudi Arabia [23] and applied the same method of assessment. A similarly high rate of obstructive sleep apnea (80.5%) was observed in a recent study among Japanese patients with type 2 diabetes [24]. A lower rate (53.2%) was observed by a more recent study published in Saudi Arabia [25] and used the Berlin scale to predict OSA, the discrepancy can be explained by the high sensitive STOP-BANG questionnaire used in the current study. The present data showed that 36.5% of patients with diabetes had daytime sleepiness with a significant expected difference between patients with diabetes and control. The present findings are higher than our previous findings [26, 27] (.22% and 6.7% respectively). In the current survey, males were elder, with longer duration of diabetes, and scored significantly higher on STOP-BANG questionnaire and coffee intake. No differences were reported regarding the daytime sleepiness score, the glycated hemoglobin, and body mass index. The higher rate of coffee intake among men can be explained by cultural factors because no differences were detected between gender regarding the daytime sleepiness which may cause high coffee consumption. The higher rate of obstructive sleep apnea among males (as predicted by STOP-BANG) may be due to age and longer duration of diabetes [28]. The effect of coffee consumption on plasma glucose is controversial [29]. The negative effects of coffee intake on insulin level, resistance, and blood glucose level seem to be genetically determined [30]. The present study showed no correlation between coffee intake and the glycated hemoglobin. Yarmolinsky et al. [9] observed the protective effects of coffee consumption on diabetes mainly through the effect on postprandial blood glucose. A recent study [31] conducted in Japan found that the severity of obstructive sleep apnea is associated with higher glycated hemoglobin especially among patients without diabetes. Interestingly the study observed that the Apnea–hypopnea index (AHI)was significantly associated with HbA1c level in total and non-diabetic individuals but not in those with type 2 diabetes mellitus. In the current study, no correlation was found between obstructive sleep apnea risk (assessed by Stop-Bang score). And the glycated hemoglobin (Data not shown). Insufficient data are present regarding the relationship between coffee intake and obstructive sleep apnea [32]. The available data showed contradicting results [9, 14, 15]. However, the anti-obesity effects and beta-cell preservation of coffee had been previously documented in obese mice [33, 34]. In the present study, no correlation was observed between coffee consumption and obstructive sleep apnea risk. It is interesting to note that, the daytime sleepiness was common among both patients with diabetes and healthy control subjects with no differences across gender. No correlation was evident between daytime sleepiness and coffee consumption. However, patients with short duration of diabetes tended to consume more coffee and a numerical value for higher coffee consumption was observed among patients with diabetes in line with Urry and colleagues from Switzerland who concluded similar observation [35]. A plausible explanation is an observation that physicians are usually advising against coffee consumption in diabetes.

### Conclusion

Patients with diabetes are at a higher risk of obstructive sleep apnea and experienced high daytime sleepiness compared to control subjects. The reverse may hold as OSA patients are more prone to diabetes mellitus. Coffee intake was negatively correlated with diabetes duration. No correlations were evident regarding coffee consumption, HbA1c, OSA, daytime sleepiness, and sex. Further multicenter studies with a larger study sample investigating the high coffee consumption observed among men and early diabetes mellitus are recommended.

## Study limitations

The results of the current survey should be viewed in the face of several limitations, namely the relatively small study sample, the reliance on questionnaires to assess OSA, and the fact that the study was conducted at a single tertiary care center. So generalization cannot be insured. Also, we did not control for smoking which may lead to OSA and affect glycemic control.

## Data Availability

All the data of the current project were presented within the manuscript.

## Abbreviations

OSA: obstructive sleep apnea
DTS: daytime sleepiness
HbA1c: the glycated hemoglobin
ESS: Epworth Sleepiness Scale
AHI: apnea hypopnea index
